# Supplementary material for: The interactome of intact mitochondria by cross-linking mass spectrometry provides evidence for coexisting respiratory supercomplexes
Source: Mol Cell Proteomics. 2017 Dec 8;17(2):216–32. doi: 10.1074/mcp.RA117.000470 (PMC5795388; doi:10.1074/mcp.RA117.000470)
Supplement: Supplemental Data [file supp_RA117.000470_133922_0_supp_23978_fzffwf.zip › spectra_annotation/mito_spectra_annotation/104-1-5-1-5-1.pdf]

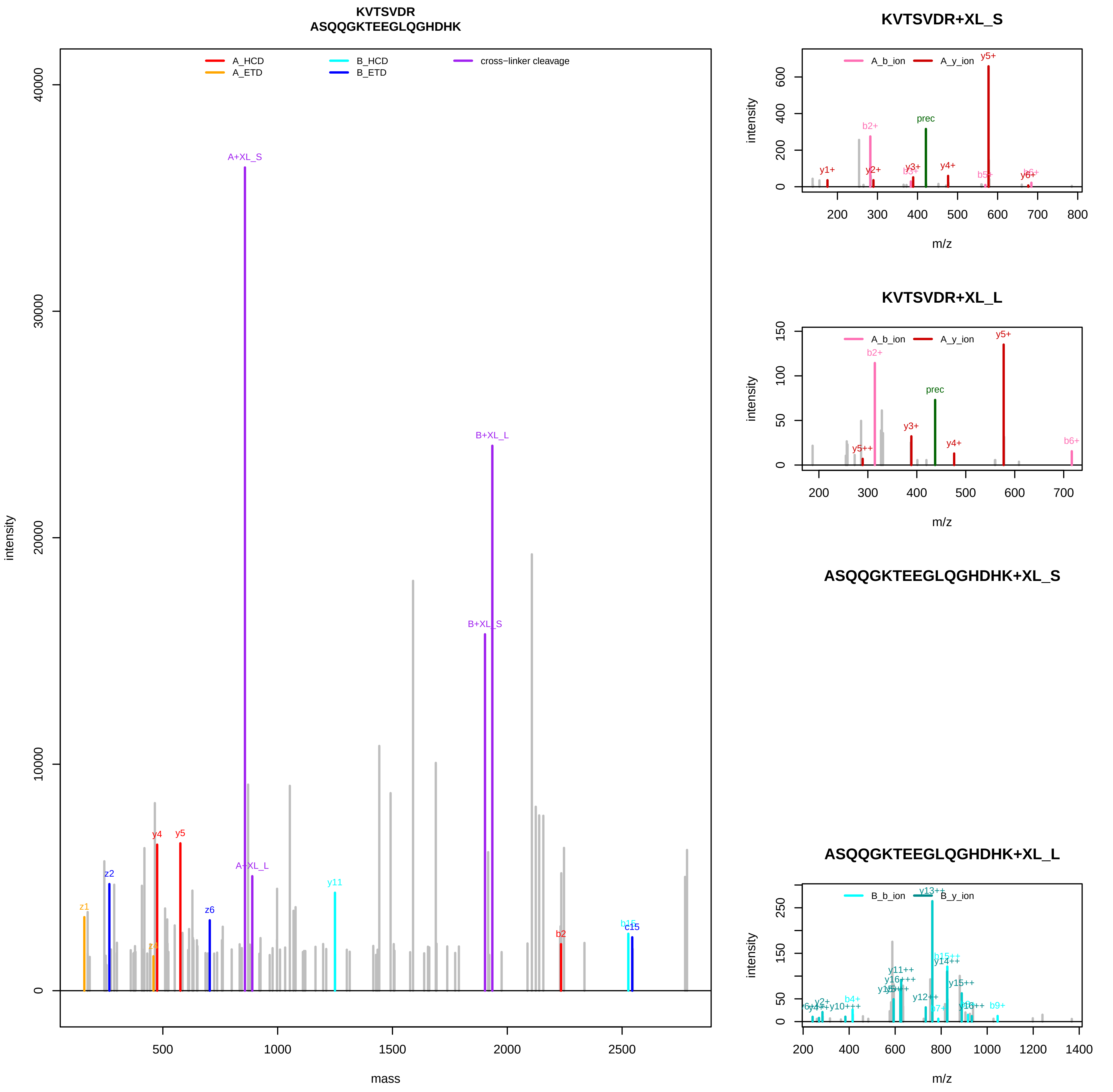
KVTSVDR
ASQQGKTEEGLQGHDHK
A_HCD
A_ETD
B_HCD
B_ETD
cross−linker cleavage
intensity
mass
A+XL_S
B+XL_L
B+XL_S
A+XL_L
y4
y5
z1
z2
z6
y11
b2
c15
KVTSVDR+XL_S
A_b_ion
A_y_ion
prec
y5+
b2+
y1+
y2+
y3+
y4+
b5+
y6+
m/z
KVTSVDR+XL_L
y5++
b6+
ASQQGKTEEGLQGHDHK+XL_S
ASQQGKTEEGLQGHDHK+XL_L
B_b_ion
B_y_ion
y13++
y11++
y16+++
y15++
y14++
y12++
y10+++
b4+
b9+
y2+
